# Spatial discontinuity of Optomotor-blind expression in the *Drosophila *wing imaginal disc disrupts epithelial architecture and promotes cell sorting

**DOI:** 10.1186/1471-213X-10-23

**Published:** 2010-02-23

**Authors:** Jie Shen, Christian Dahmann, Gert O Pflugfelder

**Affiliations:** 1 Dept of Entomology, China Agricultural University, 100094 Beijing, China; 2 Institute of Genetics, Johannes Gutenberg University Mainz, 55128 Mainz, Germany; 3 Max Planck Institute of Molecular Cell Biology and Genetics, 01307 Dresden, Germany

## Abstract

**Background:**

Decapentaplegic (Dpp) is one of the best characterized morphogens, required for dorso-ventral patterning of the *Drosophila *embryo and for anterior-posterior (A/P) patterning of the wing imaginal disc. In the larval wing pouch, the Dpp target gene *optomotor-blind *(*omb*) is generally assumed to be expressed in a step function above a certain threshold of Dpp signaling activity.

**Results:**

We show that the transcription factor Omb forms, in fact, a symmetrical gradient on both sides of the A/P compartment boundary. Disruptions of the Omb gradient lead to a re-organization of the epithelial cytoskeleton and to a retraction of cells toward the basal membrane suggesting that the Omb gradient is required for correct epithelial morphology. Moreover, by analysing the shape of *omb *gain- and loss-of-function clones, we find that Omb promotes cell sorting along the A/P axis in a concentration-dependent manner.

**Conclusions:**

Our findings show that Omb distribution in the wing imaginal disc is described by a gradient rather than a step function. Graded Omb expression is necessary for normal cell morphogenesis and cell affinity and sharp spatial discontinuities must be avoided to allow normal wing development.

## Background

The concept of Dpp as morphogen in early wing development owes much to the observation of nested target gene expression domains, initially described for *spalt *(*sal*) and *omb *[1,2] and subsequently for *vestigial *(*vg*) and the *vg *quadrant enhancer [3]. Dpp spreads from its expression domain along the A/P compartment boundary to receiving cells forming a gradient which directs patterning and growth of the wing pouch [4-6]. Dpp signaling represses the transcriptional repressor gene *brinker *(*brk*), which is thereby expressed in a gradient inverse to the Dpp signaling activity [7]. The relationship between target gene expression and Brk level is not simply reciprocal. For instance, high level *sal *expression in the central wing pouch requires direct Dpp signaling in addition to repression of *brk*, i. e. the *sal *expression domain is specified by opposing gradients [8-10]. Moreover, different target genes appear to be repressed by Brk through different mechanisms [11]. Irrespective of the mechanistic details, the nested expression pattern of *sal *and *omb *forms the basis of the threshold model of Dpp (or rather Brk) target gene regulation [12,13].

Apart from setting up gene expression patterns, the Dpp gradient appears to fulfil additional roles. Dpp is required for establishing a density gradient of the apical microtubule web (AMW), a specialization of the columnar wing pouch epithelium [14,15]. Clonal reduction of Dpp signaling in the disc main epithelium leads to wing size reduction and JNK-dependent cell death [16-19]. Mutant cells cluster into cysts in which apical and baso-lateral contacts to neighbouring wild type cells are disrupted [15,20,21]. These changes are not secondary to the activation of the JNK-pathway and apoptosis (which are elicited at the junction of cells strongly differing in Dpp signaling activity [17]) because they occur when these processes are inhibited. The mutant cysts can survive to the adult stage and differentiate. One recognized feature of cytoskeletal reorganization in *tkv *clones is the loss of the AMW. Taken together, these findings led to the hypothesis that the Dpp gradient ensures correct cell morphogenesis which is necessary for epithelial integrity [15,20]. Clones mutant for the Dpp signal transducer *Mothers against dpp *(*Mad*) lose the AMW and are extruded from the epithelium. Clone extrusion is suppressed in *mad brk *double mutant clones, suggesting that this function of Dpp is mediated by Brk-mediated Dpp target genes [15,20,21]. As the Dpp target gene *omb *is required to maintain normal epithelial structure at the A/P boundary [22,23], *omb *might be, and here is demonstrated to be, one of the mediators downstream of Dpp signaling in the establishment of epithelial architecture also elsewhere in the wing disc.

The *Drosophila *wing imaginal disc is subdivided into an anterior and a posterior compartment. In analogy to other systems, the segregation of cells at the A/P boundary is thought to be due to compartmental differences in cell-cell affinity (cell affinity hypothesis [24,25]), although recent analysis indicates an important role for increased mechanical tension for the maintenance of the A/P boundary [26]. Omb cooperates with Hh signaling to promote cell segregation at the A/P boundary, presumably by regulating the expression of cell affinity molecules [27].

Omb is essential for wing development and is sufficient to initiate secondary wing morphogenesis when ectopically expressed in the notum anlage of the wing disc [28]. The homologous vertebrate Tbx2 subfamily genes (Tbx2-Tbx5) are also involved in limb development and cause inherited haploinsufficiency syndromes associated with limb defects when mutated in humans (TBX3-TBX5) [29-31]. Tbx2 and Tbx3 are amplified or overexpressed in a wide range of neoplasms. Increased levels of TBX2/3 contribute to cancer progression by suppressing cellular senescence and by promoting invasiveness (reviewed in [32]). The latter phenomenon may be related to the morphogenetic role of Omb which we describe here.

The nested expression patterns of Sal and Omb are generally taken to support a threshold model of Dpp (or rather Brk) target gene regulation, e.g. [1,2,8,10,12,13,33,34]. We show that, contrary to prior interpretations of *omb *enhancer trap patterns, the spatial distribution of Omb is not described by a step function but rather decays smoothly toward the periphery of the wing pouch. The graded Omb distribution precludes the occurrence of spatial Omb concentration discontinuities which cause abnormal cell shape and cell extrusion in the wing disc epithelium. Moreover, the Omb gradient appears to be required to specify a gradient of cell affinity along the A/P axis.

## Results

### Omb is expressed in a gradient

**Figure 1:**
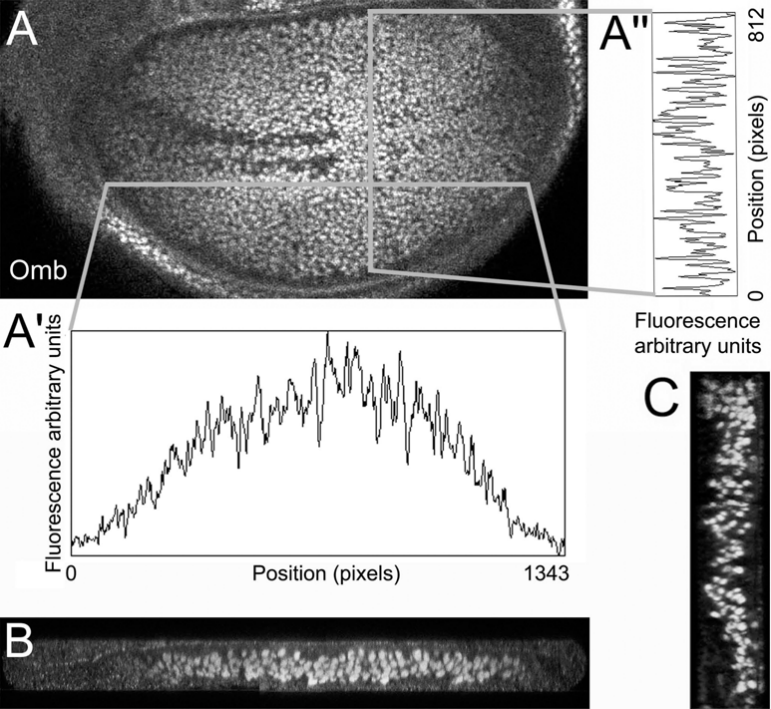
**Omb expression is graded in the larval wing pouch**. In this and subsequent figures, x-y scans are oriented with anterior left and dorsal up, x-z scans with apical up. (A) x-y section of wild type wing imaginal disc stained with anti-Omb. (A') Profile of fluorescence intensity along the A/P axis. (A'') Profile of fluorescence intensity along the P/D axis. (B) x-z section along the A/P axis. (C) x-z section along the P/D axis.

**Figure 2:**
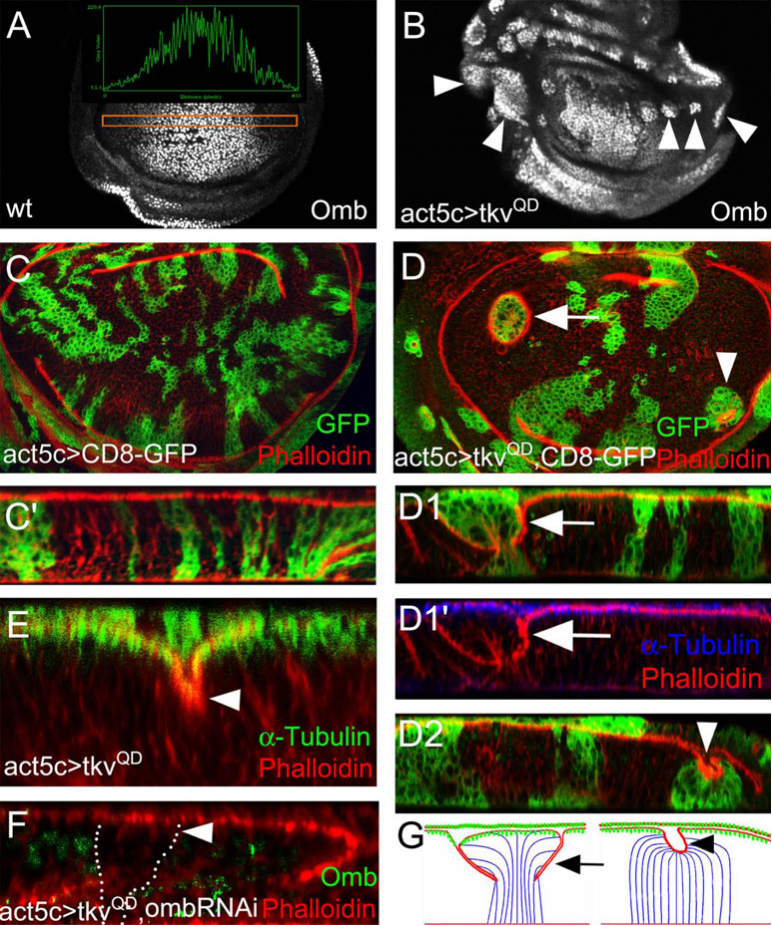
**Cellular retraction associated with lateral *tkv*^*QD *^clones**. (A) Wild type Omb expression pattern. The inset shows the profile of fluorescence intensity in a stripe of cells (orange box) along the A/P axis. (B) Lateral *tkv*^*QD *^clones (arrowheads) up-regulate Omb to a level comparable to central endogenous Omb. (C) *CD8-GFP *control clones, labelled by anti-GFP staining (green), show normal phalloidin staining. (C') x-z scan through the same disc. (D) Lateral *tkv*^*QD *^clones, labelled by CD8-GFP co-expression (green), either form a fold at the clonal border (arrow) or retract cells toward the basal side within the clone (arrow head) as revealed by phalloidin staining. (D1 and D2) x-z scans through clones marked by arrow and arrowhead, respectively, in (D). The x-z sections presented in C', D1, D1', and D2 are derived from C and D, respectively, but are shown at a 1.5-fold higher magnification. D1 shows the retraction of cells at the clonal border and D1' shows loss of the apical microtubule web in retracted cells (arrow). (E) x-z scan through *tkv*^*QD *^clones. The apical microtubule web, stained by anti-α-tubulin (green), is reduced in the retracting cells (arrowheads). (F) x-z scan of *UAS-tkv*^*QD *^*UAS-ombRNAi *clone. Cell shape in this lateral clone (arrowhead) which is identified by the lack of Omb appears normal. The x-y position of the clone is shown in Additional File 2B. (G) Model of cell shape changes in clones with peripherally (left) and centrally (right) retracting cells. High local microtubule density (shown in green) is found both in the peripodial membrane (squamous epithelium) and the AMW of the underlying columnar epithelium. Mutant cells are rendered with blue, wild type cells with black outlines. The left cartoon visualizes that cellular retraction at the clone boundary also leads to non-autonomous attenuation of the AMW in the flanking wild type cells. Phalloidin staining is red in all panels.

*omb *expression in the wing imaginal disc is frequently visualized indirectly by monitoring the activity of enhancer trap insertions in the *omb *locus. As will be discussed below, the spatial patterns of such indirect read-outs are likely to differ in detail from that of endogenous Omb. We, therefore, analyzed the Omb distribution directly by immunofluorescence using an antibody specific to Omb (Fig. 1A; Additional File 1). In the wing disc pouch of late-third instar larvae, Omb was high in the center and declined smoothly toward the lateral (anterior and posterior) margins of the pouch (Fig. 1A' and Fig. 2A). This lateral decline was also conspicuous in x-z confocal sections parallel to the A/P axis (Fig. 1B). Along the orthogonal proximo-distal (P/D) axis, the Omb distribution was not noticeably graded (Fig. 1A'', C). Omb distribution was also graded along the A/P axis in the wing disc pouch of early-third instar and mid-third instar larvae (Additional File 1C-E), suggesting that the distribution of Omb is graded along this axis throughout the third instar larval stage. In the commonly used *omb-lacZ *enhancer trap line *omb*^*P1 *^[35], β-galactosidase, as visualized by immunofluorescence, was also expressed in a gradient, indicating that the Omb gradient does not arise by post-transcriptional regulation (Additional File 1A). To rule out that unspecific binding of the polyclonal anti-Omb antiserum contributed to the graded appearance of the Omb expression profile, we expressed *omb-RNAi *in the *en-Gal4 *domain of the wing disc (Additional File 1B). Such discs showed very low staining in the posterior compartment indicating high specificity of the antiserum used. Since RNAi does not completely eliminate Omb expression the actual specificity will be even higher, as indicated by the lack of staining in parts of the notum region (Additional File 1A-E).

In order to determine whether the graded distribution of Omb is required for the correct architecture of the wing disc epithelium, three types of mosaic clones were generated, all causing disruptions of the smooth Omb concentration profile. In the first, Omb was induced laterally to a level comparable to the endogenous peak concentration. In the second, Omb was reduced, and in the third, Omb was overexpressed beyond the maximum endogenous level. In mosaic discs, cytoskeleton and apico-basal organization were visualized by phalloidin staining (specific for filamentous actin), anti-α-tubulin staining (revealing the AMW), and anti-DE-cadherin immunofluorescence (revealing adherens junctions [36]).

### Expression of constitutively active Tkv causes cell retraction in the lateral wing disc

Expression of a constitutively active form of the type I Dpp receptor Thickveins (Tkv^QD^, [2]) in *act5C>tkv*^*QD *^clones increased Omb to a level comparable to its peak endogenous level (Fig. 2B and Additional File 2A''). In the lateral region, clones rounded up and accumulated F-actin either at the clonal border (Fig. 2D, arrow) or within the clone (Fig. 2D, arrowhead). This behaviour was not observed in control clones (Fig. 2C). When inspected in the x-z plane, cells in the clone were either shortened at the clonal border (Fig. 2D1, arrow) or in the center of the clone (Fig. 2D2, arrowhead). We use the term retraction to describe this shortening which leads to an apical indentation while basally the arrangement of clonal cells appears not to differ from the surrounding cells. The position of the apico-basal retraction appeared to be governed by clone size. Large lateral clones rounded up and exhibited retracting cells at the clonal border, thereby becoming surrounded by a circular fold (arrows in Fig. 2D and 2D1, arrowheads in Additional File 3A and 3A'). Small lateral clones rounded up and retracted toward the basal membrane in the clonal center (arrowheads Additional File 4B and 4B'). Unlike extruding *tkv *mutant clones [15,20], *act5C>tkv*^*QD *^clones maintained apical contact to neighbouring cells. These results suggest that up-regulated Dpp signaling in the lateral region of the wing pouch is sufficient to change the shape of epithelial cells if these are in contact with cells experiencing less Dpp signaling activity.

The wing pouch AMW depends on Dpp signaling. AMW density is graded along the A/P axis with a broad maximum in the center of the pouch and attenuation laterally [14] (Additional File 4). In clones lacking Tkv activity, the AMW is strongly reduced [15,20]. However, the AMW was also lost in retracted cells both inside and outside of *UAS-tkv*^*QD *^clones (Fig. 2D1' and 2E), suggesting that its presence depends on the spatial continuity of Dpp signaling. To test whether it is the up-regulated Omb level, elicited by increased Dpp signaling (Fig. 2B), which causes the retraction of cells in the lateral wing pouch, *UAS-ombRNAi *was co-expressed with *UAS-tkv*^*QD*^. OmbRNAi essentially eliminated Omb under these conditions. Furthermore, the retraction of lateral cells was prevented by this regime (Fig. 2F and Additional File 2B). Thus, the up-regulation of Omb is required to mediate the re-organization of the epithelial architecture in lateral *act5C>tkv*^*QD *^clones.

### Lack or strong reduction of Omb causes retraction of cells in the wing pouch

**Figure 3:**
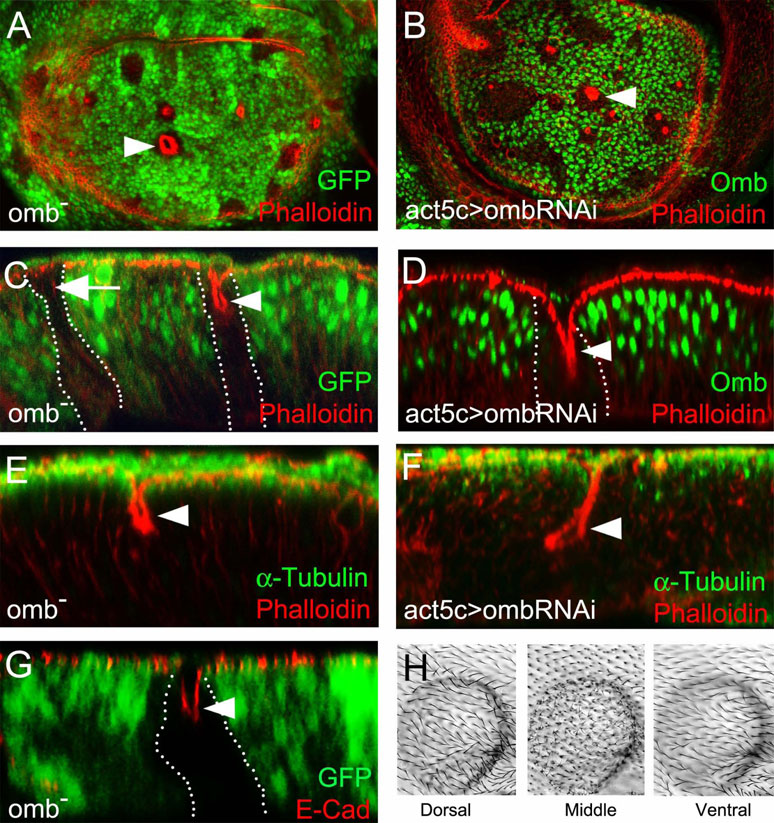
**Lack of Omb causes cells to retract toward the basal side**. (A) *omb *null mutant clones (arrowhead), labelled by absence of GFP, contain retracted cells in the clonal center. (B) *ombRNAi *clones (arrowhead), labelled by absence of *Omb*, also contain retracted cells in the clonal center. (C-G) x-z scans of *omb *null mutant clones (marked by absence of GFP) and *ombRNAi *clones (marked by reduction of Omb). (C) Central (arrowhead) but not the very lateral (arrow) mutant clones contain retracted cells. (D) *ombRNAi *clone (marked by reduced anti-Omb staining, arrowhead) with strong central retraction toward the basal lamina. (E and F) Reduction of the apical microtubule web in retracting cells of *omb *null mutant clone (arrowhead, E) and *ombRNAi *clone (arrowhead, F). (G) The DE-Cadherin level appears normal (arrowhead) in *omb *null mutant clones (absence of GFP). (H) Dorsal, middle, and ventral views of an *omb *mutant clone in adult wing.

As a second test for determining the role of Omb in the maintenance of epithelial architecture, *omb *loss-of-function clones were generated. In the wing pouch, small *omb *mutant clones rounded up and accumulated F-actin in the clonal center (Fig. 3A). (F-actin staining can appear annular when the disc is optically sectioned close to the apical surface, cf. Fig. 3C-2G). When visualized in the x-z plane, the apico-basal retraction of mutant cells was apparent in the center of the clone (Fig. 3C, arrowhead). No retraction was seen in clones of the lateral periphery (Fig. 3C arrow). In the retracted clonal cells, the AMW was strongly reduced (Fig. 3E). Staining against DE-cadherin showed that retracting cells retained apical contact among themselves and to the surrounding phenotypically wild type cells (Fig. 3G). The close proximity of E-cadherin-labelled adherens junctions of neighboring cells, as seen in x-z sections, indicates that retracting cells were apically constricted. Distinct apicolateral junctions were more easily discernable in shallow retractions (cf. Additional File 5C, C'). To confirm the observations obtained with *omb *mutant clones, *act5C>ombRNAi *clones were generated. In these, Omb was strongly reduced (Additional File 6). *Act5C>ombRNAi *clones showed the same phenotype as *omb *null mutant clones (Fig. 3B, D, F). These results indicate that Omb is required to maintain the correct shape of epithelial cells in the larval wing pouch.

JNK-dependent cell death has been observed previously on either side of the border of clones disrupting the normal Dpp signaling gradient [17]. Cellular retraction in *omb *clones appeared independent of cell death. Although caspase-3 positive cells were occasionally observed at the border of clones (Additional File 5A, A' arrow), many retracting clones showed no evidence of cell death (Additional File 5A-C, arrowheads) and still proliferated (Additional File 5D and 5E, arrowheads). The relationship between cellular retraction and JNK-dependent cell death was analyzed in more detail for Omb-overexpressing clones (see below). In the adult wing, the majority of *omb *clones manifested as clustered microchaetae restricted to either the dorsal or ventral leaflet, similar to the predominant phenotype of *UAS-tkv*^*QD *^clones. Occasionally, *omb *clones survived to adulthood as cyst-like structures located between the dorsal and ventral wing surfaces indicating complete retraction of mutant cells during the pupal stage. In both cases, retracting cells survived up to the stage of cuticle deposition (Fig. 3H).

### Overexpression of *omb *causes cellular retraction in both autonomous and non-autonomous ways

**Figure 4:**
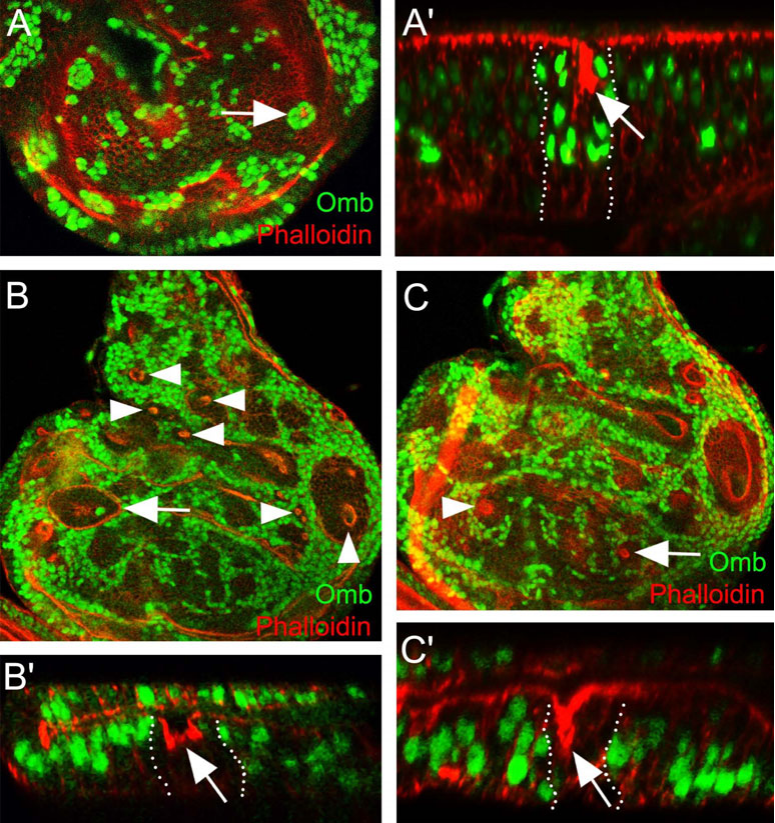
**Omb overexpression causes autonomous and non-autonomous cellular retraction**. *Tubα1>omb *clones are marked by strong anti-Omb staining (green). (A) Omb overexpressing cells tend to scatter in the epithelium. Thus, mosaic discs generally contain few cases of grouped cells (arrow). These show central retractions. (A') x-z scan through a retracting clone. (B and C) Enhanced generation of Omb overexpressing cells by more severe heat shock conditions causes clustering of wild type cells into groups with smooth outlines (arrowheads). In these non-clonal wild type cell groups, cellular retraction occurrs in the periphery (B') or in the center (C'). (A-C) are x-y scans, (A'-C') x-z scans. Arrows in A to C indicate the cell clusters that are shown in A' to C'.

In the third strategy, direct Omb over-expression in *tubα1>omb *clones led to local Omb accumulations that strongly exceeded the peak level of the endogenous protein. Mutant clones showed cellular retraction in the center of the clone (Fig. 4A and 4A'). When clone frequency was enhanced by increasing the heat-shock temperature, wild type cells were clustered into groups with smooth outlines surrounded by Omb-overexpressing cells. In these non-clonal wild type cell groups, cellular retraction occurred either in the periphery (Fig. 4B') or in the center (Fig. 4C'). Judged by the continuity of apical phalloidin staining, these retracting wild type cells maintained apical contact with neighboring cells overexpressing *omb*. This situation corresponds morphologically to *omb *loss-of-function clones surrounded by wild type cells, where cells with reduced Omb undergo apico-basal contraction. The data show that overexpression of *omb *can cause cellular retraction both autonomously and non-autonomously. *Omb *overexpression is known to induce JNK-dependent apoptosis [17] (Additional File 7A). If apico-basal retraction were a consequence of the initiation of apoptosis, then blocking JNK pathway activation and apoptosis should block cellular retraction. Repression of cell death by a dominant negative form of JNK (Bsk^DN^, [37]) or by co-overexpression of P35 [38] did not rescue the cellular retraction phenotype (Additional File 7) suggesting that cellular retraction is not coupled to execution of the apoptosis pathway.

### Omb affects cell affinity in a concentration-dependent manner

**Figure 5:**
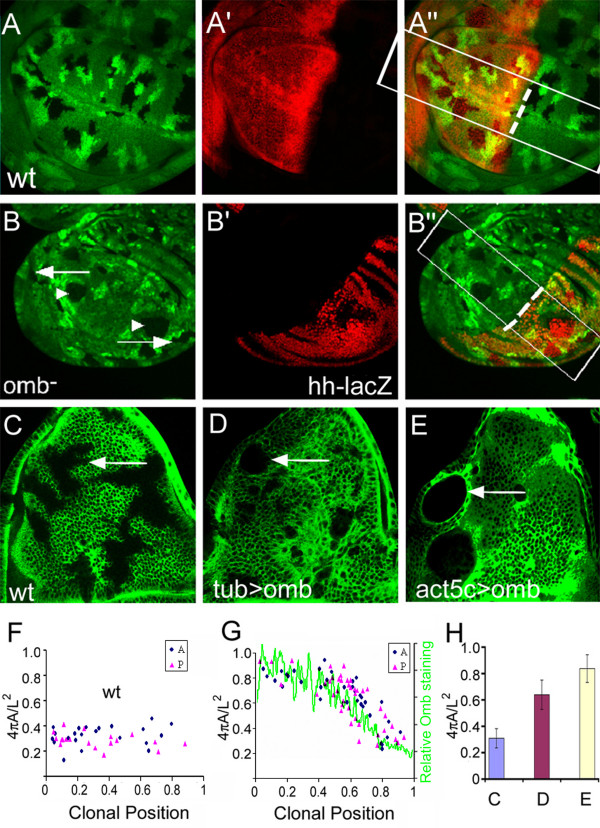
**Omb regulates cell affinity in a concentration-dependent manner**. Only Clones within a region corresponding to the boxed areas in A'' and B'' were selected for measurement. The position of the A/P boundary (broken line) was determined by Ci (A') or *hh-lacZ *(B') staining. Area (A) and perimeter (L) of clones were determined. For calculation of the shape factor, the formula 4ΠA/L^2 ^was used. Clonal position relative to the A/P boundary was determined by measuring the distance between the center of the clone and the A/P boundary normalized to the distance from the edge of the wing imaginal disc to the A/P boundary. (A) wt control clones (generated in *hs-flp hs-GFP FRT19/FRT19 *larvae) were wiggly independent of their position. (B) *l(1)omb*^*D4 *^clones (generated in *hs-flp hs-GFP FRT19/l(1)omb*^*D4 *^*FRT19 *larvae) were round when close to the A/P boundary but wiggly in the periphery. (C-E) flip-out clones were generated by heat-shocking *act5C>CD2>Gal4 *(C), *tubα1>CD2>Gal4, UAS-omb *(D), and *act5C>CD2>Gal4, UAS-omb *(E) flies (all containing *hs-flp*^*22 *^on the first chromosome). Clones were visualized by lack of CD2 staining. Larvae were reared at 18°C to reduce the dispersal of Omb-overexpressing cells. (F and G) Shape factor plotted as a function of clonal position. Clonal position value is "0" at the A/P boundary and "1" at the edge of the wing disc. A and P clones are represented by blue dots and red triangles, respectively. In (G), the decay of the Omb gradient, measured in a different wing disc, is shown as a green line. (H) Average shape factor of notum clones expressing no (blue), low (purple) or high (yellow column) Omb. The difference in shape factor was pairwise statistically significant (p < 0.001).

We noticed that central *omb *mutant clones in which apico-basal retraction could be observed were of roundish shape (arrowhead in Fig. 3A) whereas non-retracting clones in the lateral periphery had wiggly contours. In the framework of the cell affinity hypothesis [24,25], differences in cell affinity are expected to influence the shape of a clone. Clones with irregular outlines are thought to have surface properties similar to their neighboring cells. The round shape and smooth border of clones on the other hand is considered to reflect differences in the affinity of cells in and outside of the clone. *omb *mutant clones located in the central region had a rounder shape and smoother borders compared to clones located in the periphery (Fig. 5B). For quantification, we calculated the shape factor (SF) of *omb *mutant clones using the formula 4ΠA/L^2 ^(A = area of clone, L = perimeter of clone) as a function of their distance to the A/P boundary [39]. A circular clone will have a SF value of 1 whereas wiggly clones will have values smaller than 1. Control clones had irregular shapes (SF = 0.31 ± 0.07) regardless of their position in the wing imaginal disc (Fig. 5F). In contrast, *omb *mutant clones in the periphery were irregular in shape (SF = 0.39 ± 0.13) whereas they had a rounder shape when located in the vicinity of the A/P boundary (SF = 0.83 ± 0.07) (Fig. 5G). Clones at intermediate positions had intermediate values, indicating that the shape of *omb *mutant clones is graded along the A/P axis. *Omb *mutant clones in the A and P compartments had similar shape factors when located at similar distance from the A/P boundary. As shown above, the expression of Omb is graded along the A/P axis of the wing pouch. The difference in Omb levels between *omb *null clones and wild type surrounding cells will be highest in the vicinity of the A/P boundary and lowest at the periphery of the wing pouch. Thus, the SF value of *omb *mutant clones correlates with the difference in Omb protein concentration between clone and the neighboring wild type cells. This suggests that the affinities of cells in peripheral and central regions of the wing imaginal disc are different and that Omb activity contributes to this difference in a concentration-dependent manner.

In a reverse approach, we expressed Omb to different levels in the notum region of the wing disc which contains little endogenous Omb [23]. Flip-out clones were generated in which *omb *was expressed under the control of *tubα1>Gal4 *or *act5C>Gal4 *(the relative strength of these Gal4 drivers as monitored by UAS-GFP expression was 1:1.8). While control clones had irregular outlines (Fig. 5C), clones expressing Omb were rounder, with a SF value that increased with Omb level (Fig. 5D, E, H).

Both experiments indicate that the level of Omb controls cell affinity and that Omb can exert this control both in its endogenous domain (pouch) as well as in an ectopic setting (notum).

## Discussion

### Dpp gradient interpretation in the larval wing pouch

Thresholds, in a strict sense, should lead to a sharp transition in gene expression from one cell to the next [40]. This is, in fact, not observed for any of the early wing pouch targets ([11,41-45]; this report). Similarly, in the well studied *Xenopus *embryo model, activin forms a gradient that initially leads to a graded distribution of the target protein Xbra which is refined to form sharp boundaries under the involvement of secondary factors [46,47]. Dpp, via Sal and Omb, specifies the highly stereotypic positions of wing veins L2 and L5 but also in this case additional genes are involved [48,49]. Recent work on gradient interpretation supports the notion that smooth gradients of a single morphogen may not suffice to specify sharp transitions in nuclear or cell specification, e. g. [44,50-53]. These findings suggest that the concept of positional information [54] may not be valid in its simplest form [13,55].

Most studies on *omb *as a Dpp target gene in wing develoment were performed with *omb-lacZ *or *omb-Gal4 *enhancer trap lines [35,56]. These lines quite faithfully render the overall *omb *expression pattern but differ from endogenous *omb *in detail (Additional File 1). Differences in the steepness of graded gene expression patterns between direct (RNA in situ hybridization or protein immunofluorescence) and indirect measurements (enhancer trap) have been noted before (e.g. Dad [41], *brk *[43]). A systematic deviation will occur when β-galactosidase is monitored by histochemical staining (e.g. with the common chromogen X-gal). β-galactosidase is a homotetrameric protein that is only active in its oligomeric form [57]. This will cause a sigmoid dependence of activity on protein concentration. Similarly, when Gal4 expression is monitored by UAS-reporter activity, synergistic binding of dimeric Gal4 to the UAS pentamer of standard pUAST derivatives [58] can cause a non-linear response [59].

### Presence of a gradient of cell affinity

Transplantation experiments in developing insect wings suggest that cells within a compartment differ in cell-cell affinity. Cells at the same proximo-distal position have a similar P/D affinity value and intermingle to form a wiggly interface. In contrast, groups of cells transplanted to different proximo-distal positions will rearrange contacts and form a roundish patch thereby minimizing contact with the surrounding tissue [60].

We tested whether a gradient of cell affinity is present along the A/P axis in the *Drosophila *wing disc by analyzing the shape of *omb *mutant clones (Fig. 5). We found that *omb *mutant clones close to the A/P boundary had smooth borders, indicating that *omb *mutant cells sort out from neighboring wild type cells. With increasing distance from the A/P boundary, clone shape became progressively irregular. This, in the framework of the cell affinity model, suggests the existence of a gradient of cell affinity which is disrupted by *omb *clones. The gradient of cell affinity correlates with the level of Omb expression, indicating that Omb, at least in part, shapes this gradient. This was confirmed by expressing Omb to different levels in a tissue with little endogenous Omb.

**Figure 6:**
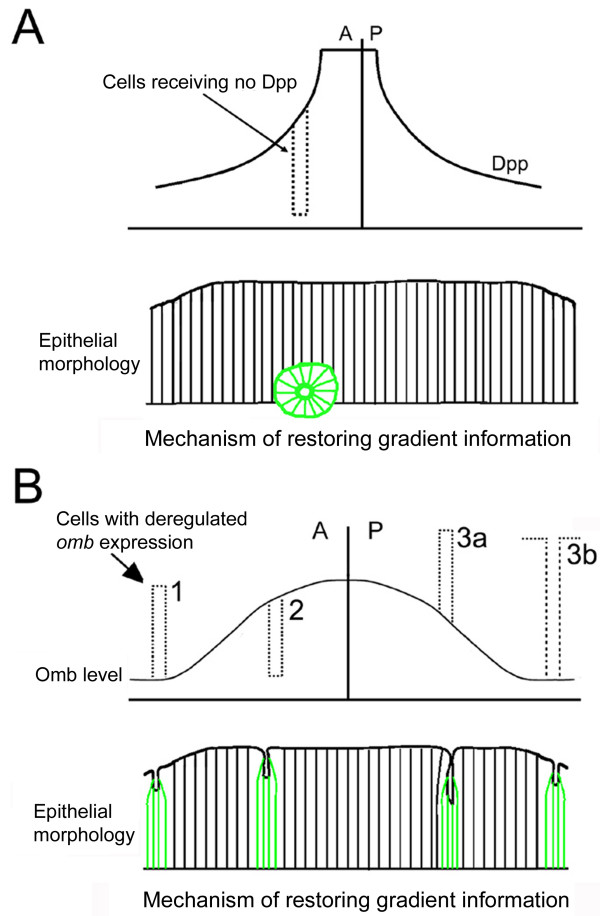
**Mechanism of restoring gradient continuity**. Clones and the ensuing discontinuities in Dpp signalling or Omb levels are symbolized by dotted contours. (A) Clones lacking Dpp signaling generate sharp discontinuities in the Dpp signaling gradient. Extrusion of mutant cells restores the monotonous decline of the gradient. (B) Manipulations of Omb level. Numbers next to the dotted bars refer to the experimental manipulation by which changes in Omb level were effected. (1) Upregulation of Omb in lateral *tkv*^*QD *^clones. (2) Loss of Omb in *omb *null mutant clones (strong Omb reduction by ombRNAi was similarly effective in eliciting cellular retraction). (3a) Clone of cells (*tub>omb*) strongly overexpressing Omb and surrounded by wild type cells. (3b) Group of wild type cells surrounded by cells strongly overexpressing Omb. In the bottom diagrams, presenting x-z views of mosaic wing discs, clonal cells are drawn in green.

Omb may not be the only transcription factor controlling an affinity gradient in the wing pouch. Clones mutant for *sal *have round borders in the central part of the disc and are wiggly in distal parts, suggesting that Sal, which is downstream of Omb in the wing pouch [61], also affects cellular affinity [62]. Given the predominantly apical defects seen in clones that differ in Omb level from the surrounding tissue, it is plausible that Omb controls the expression of apically located cell adhesion molecules. In the simplest case, the Omb-controlled affinity gradient will be similarly shaped as the Omb gradient but an inverse gradient cannot be ruled out. In both cases, local disruption in the Omb level would lead to changes in the spatial distribution of affinity molecules causing clones to round up and, in the extreme case, to sort out of the epithelium (Fig. 6). Graded expression of Omb may play a related role in setting planar polarity values in the development of the adult abdominal segments [63].

### The importance of being graded

In addition to Dpp itself, several gene products directly or indirectly downstream of Dpp are expressed in a graded manner (e. g. Tkv, Brk, Dad, Sal, Omb, Capricious (Caps) and Tartan (Trn)). Some of these are part of the Dpp signaling cascade (Tkv, Brk, Dad), Sal and Omb are nuclear effectors, Caps and Trn cell surface proteins. Furthermore, the density of the AMW is graded along the A/P axis. In *tkv* clones, the AMW is lost [15,20]. We show here, that in and around large *UAS-tkv*^*QD *^clones, in which the Dpp pathway is constitutively active, the AMW is also strongly reduced in retracting cells but not in central non-retracting cells (Fig 2D1'), indicating that the reduction in AMW density is elicited by the apposition of cells strongly differing in Dpp signaling acitvity. AMW reduction in *tkv*^*QD *^clones indicates that AMW density is not only controlled by the Dpp level but is also subject to control which is levied by Dpp signaling discontinuities.

Dpp is required for wing disc growth and proliferation [1,16,64]. The uniform proliferation across the wing disc has been difficult to reconcile with the exponential shape of the Dpp gradient and with the finding that ubiquitous expression of Dpp or of Dpp pathway components can promote overgrowth (reviewed in [65]). According to a model proposed by Rogulja and Irvine, two Dpp-dependent growth promoting systems coexist in the wing imaginal disc, only one of which is responsive to the gradient of Dpp signaling [65]. More recently, Basler and colleagues argued, that a gradient of Dpp signaling is not required for wing growth [66]. In the latter model, Dpp requirement differs qualitatively for growth and patterning.

**Figure 7:**
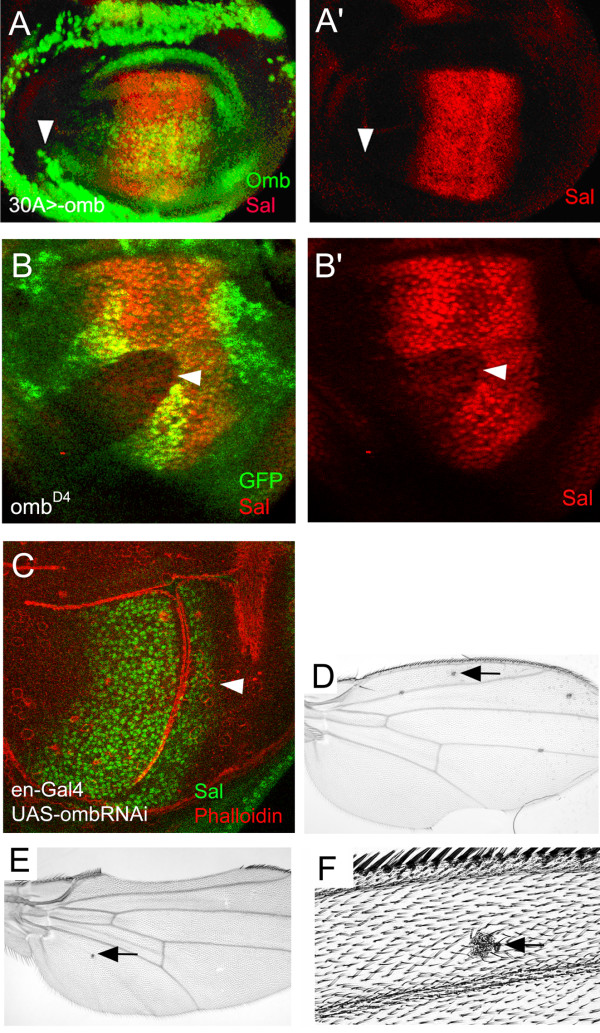
**Epithelial effects of Omb misexpression are not mediated by Sal**. (A, A') Strong ectopic Omb has little effect on Sal expression. The arrowhead points to where the Gal4 30 expression domain [58] overlaps the wing pouch. (B, B') Complete loss of *omb *(in a *l(1)omb*^*D4 *^clone, arrowhead) leads to strong reduction of Sal. (C) Incomplete elimination of *omb *function (in *en>ombRNAi*) only slightly decreases Sal expression. The arrowhead points to the posterior compartment, separated from the anterior compartment by a deep fold due to posterior reduction in Omb [23]. (D-F) The phenotype of *omb *l-o-f clones (arrows) is elicited also outside the Sal domain. Cuticular manifestations of retraction and extrusion events were found anterior to L2 (D, E) and posterior to L5 (E).

To what extent are genes known to be regulated by Dpp involved in apico-basal retraction? We show that spatial discontinuity in Omb level is necessary for this phenotype. Central loss-of-function clones of *sal*, which is expressed in a gradient with a shorter A-P width than *omb *[2,42,44], were reported to sort out of the surrounding epithelium indicating that Sal, too, is required to maintain epithelial integrity [62]. Omb is known to be required for *sal *expression [61], (Fig. 7B). This raises the question of whether Omb acts via *sal*. We do not think that the effect of *omb *l-o-f needs to be mediated by *sal*. First, *ombRNAi *is sufficient to elicit cellular retraction (Fig. 3B, D, F) but does not cause loss of Sal expression (Fig. 7C). Second, retraction caused by *omb *g-o-f is not mediated by *sal *because its expression is not induced by ectopic Omb (Fig. 7A). Third, in the adult wing, we observed the cuticular manifestations of retraction and extrusion events also anterior to longitudinal vein L2 and posterior to L5 (L2 forms in the steep anterior slope of the Sal expression domain, L5 posterior to the Sal domain [67]), indicating that these retractions did not arise as a consequence of secondary local Sal reduction (Fig. 7D-F)). Clones lacking Dpp signaling are *extruded *from the wing disc epithelium [15,20]. This does not occur with *sal *[62] or *omb *mutant clones during larval development, and only (to a limited extent) during pupal development. With regard to extrusion from the larval wing imaginal disc there is, thus, a qualitative difference between *tkv *and *omb/sal *clones. We surmise that Dpp target genes other than *omb *and *sal *are involved in generating the *tkv *mutant extrusion phenotype.

Graded gene expression appears required also along the orthogonal dorso-ventral (D/V) axis. Vestigial is expressed in a symmetrical gradient that decays away from the D/V boundary [68] and is required for patterning and growth control along the D/V axis. Vg gain-of-function clones induce JNK at the clone border which is more remote from the D/V boundary, indicating that JNK is activated by spatial discrepancy in Vg levels [17]. Such clones (and their wild type twin spots) become larger with increasing distance from the D/V boundary and retract from the apical epithelial surface [69]. Apparently, the creation of local discontinuities in Vg level leads to increased proliferation on both sides of the clonal border. The importance of a graded Vg distribution is underlined by the reduced size of both *vg *mutant and Vg overexpressing wings [70]. A similar requirement for graded gene expression to ensure normal wing disc proliferation was shown for *dachsous *and *four-jointed *[71,72]. Like clones mutant for factors downstream of DPP, which disrupt A/P-boundary-centered gradients, *vg *mutant clones, in which JNK-mediated apoptosis is suppressed, are extruded from the wing disc [70]. Retraction and extrusion, like morphogenetic apoptosis [17] may be universal mechanisms for correcting disturbances in the graded expression of factors required for patterning and growth of the wing disc epithelium. The gradient of cell affinity may serve to stabilize patterns of positional information against fluctuations of the respective morphogen activity gradients.

## Conclusion

In the field of developmental biology, positional information and morphogens are important concepts to understand how cellular fields can be patterned. The *Drosophila *wing imaginal disc is a well studied system in which the diffusible protein Decapentaplegic, expressed in a stripe along the anterior-posterior compartment boundary, leads to the nested expression of target genes (*spalt, omb, vestigial*). The nested expression patterns are thought to arise from different thresholds of gene activation. We show by quantitative analysis that Omb expression is graded along the anterior-posterior axis. Manipulations that introduce spatial discontinuity in the Omb level cause disruptions of epithelial morphology, indicating that the normal graded distribution of Omb is important for proper wing development. We furthermore provide evidence that the Omb gradient instructs the formation of a gradient of cell affinity which may reduce cell mixing in the compartment.

## Methods

### *Drosophila *stocks

Stocks are described at http://flybase.bio.indiana.edu unless indicated otherwise. *l(1)omb*^*D4 *^and *l(1)omb*^*3198 *^were used as *omb *null alleles [73]. Transgenes: *UAS-CD8-GFP, tubα1>CD2>Gal4, act5c>CD2>Gal4, UAS-tkv*^*QD*^, *UAS-ombRNAi-C4 *[23], *UAS-omb 4-3 *[28], *UAS-bsk*^*DN *^[37] and *UAS-p35 *[38]. Enhancer trap lines: *hh*^*P30 *^[74] and *omb*^*P1 *^[35]. Larvae were reared at 25°C or at the indicated temperature.

### Clone generation

Marked clones of mutant cells were generated by Flp-mediated mitotic recombination [75] subjecting first or second instar larvae to a 36-39°C heat-shock for 30 min. Transgenes were expressed using the Gal4-UAS system [58]. The larval genotypes for clone generation were as follows:

1. *tkv*^*QD *^clones: *y w hsp70-Flp; act5c>CD2>Gal4/UAS-tkv*^*QD*^

2. *CD8-GFP *clones: *y w hsp70-Flp; UAS-CD8-GFP; act5c>CD2>Gal4/UAS-CD8-GFP*

3. *tkv*^*QD *^*CD8-GFP *clones: *y w hsp70-Flp; UAS-CD8-GFP; act5c>CD2>Gal4/UAS-tkv*^*QD*^

4. *tkv*^*QD *^*ombRNAi *clones: *y w hsp70-Flp; UAS-ombRNAi; act5c>CD2>Gal4/UAS-tkv*^*QD*^

5. *omb *clones: *y w hsp-GFP hsp70-Flp FRT19/omb*^*3198 *^*FRT1 *and *y w hsp-GFP hsp70-Flp FRT19/omb*^*D4 *^*FRT19*

6. *UAS-ombRNAi *clones: *y w hsp70-Flp; act5c>CD2>Gal4/UAS-ombRNAi *and *y w hsp70-Flp; UAS-ombRNAi; act5c>CD2>Gal4*

7. *UAS-omb *clones: *y w hsp70-Flp; act5c>CD2>Gal4/UAS-omb *and *y w hsp70-Flp; tub>CD2>Gal4; UAS-omb*

8. *UAS-omb UAS-p35 *clones: *y w hsp70-Flp; UAS-p35; act5c>CD2>Gal4/UAS-omb *and *y w hsp70-Flp/UAS-p35; act5c>CD2>Gal4/UAS-omb*

9. *UAS-omb UAS-bsk*^*DN *^clones: *y w hsp70-Flp/UAS- bsk*^*DN *^; *act5c>CD2>Gal4/UAS-omb*

10. wildtype control clones: *y w hsp-GFP hsp70-Flp FRT19/FRT19 *and *y w hsp70-Flp; act5c>CD2>Gal4*

### Omb-antiserum

Omb-antiserum, first mentioned in a footnote in [28], was raised against His-tagged full-length Omb protein expressed from the bacterial vector pET15b (Novagen, Darmstadt, Germany) and purified by Ni^2+ ^chelate chromatography. Rabbits were immunized by sub-cutaneous, mice by intraperitoneal injection of the antigen along with antibody multiplier (Linaris, Wertheim, Germany).

### Immunohistochemistry

Imaginal discs dissected from third instar larvae were fixed and stained with rhodamine phalloidin (Invitrogen, Carlsbad, CA, USA) and the appropriate primary antibodies: Rat anti-Ci 2A1, 1:4 (gift from R. Holmgren, Northwestern University, Evanston, IL, USA), mouse anti-CD2 (1:2000) (Serotec, Oxford, UK), rabbit anti-Omb (1:1000), rabbit anti-GFP (1:2000) (Clontech, Mountain View, CA, USA), rabbit anti-cleaved-Caspase-3 (1:200) (Cell Signaling, Danvers, MA, USA), rabbit anti-PH3 (1:200) (Upstate Biotechnology, Lake Placid, NY, USA), rabbit anti-β-galactosidase, 1:2000 (Cappel, Abnova, Heidelberg, Germany), goat anti-DE-cadherin (1:200) (Santa Cruz, Santa Cruz, CA, USA), and mouse anti-α-tubulin (1:1000) (Sigma, Munich, Germany). Secondary antibodies used were: Anti-mouse FITC, anti-mouse Cy5, anti-rabbit FITC, anti-rabbit Cy5, and anti-goat Cy3 (1:100, Jackson Immuno Research. West Grove, PA, USA). Images were recorded on a confocal microscope. The plot profile of anti-Omb staining was measured using the Image-J program (NIH, Bethesda, MD, USA).

### Imaginal disc cryosections

After secondary antibody staining, discs were re-fixed for 30 min in 4% paraformaldehyde, washed, and stored in 30% sucrose at 4°C overnight. Discs were oriented in Tissue-Tek (Sakura Finetek, Torrance, CA, USA), frozen and cut into 25 μm sections on a cryostat (Cryo-Star HM 560, Microm).

### Clonal shape measurement

For determination of position and shape factor of clones, the A/P boundary was determined by Ci or *hh-lacZ *staining, area (A) and perimeter (L) of clones were measured and the shape factor (4ΠA/L^2^) was calculated. The clonal position relative to the A/P boundary was determined by measuring the distance of the center of the clone to the A/P boundary divided by the distance from the edge of the wing imaginal disc to the A/P boundary

## Authors' contributions

JS, CD, and GOP conceived and designed the experiments which were performed by JS. Data were analyzed by JS, CD, and GOP. JS and GOP wrote the manuscript. All authors read and approved the final manuscript.

## Supplementary Material

Additional file 1 **Difference in the steepness of graded expression between Omb immunofluorescence and the *omb-lacZ *enhancer trap line *omb*^*P1*^**. (A) *omb*^*P1 *^disc double stained with anti-Omb (A') and anti-β-galactosidase (A''). The fluorescence intensity distributions in stripes of cells (orange boxes) along the A/P axis were measured using the Image-J program and are shown in a-a". (B) *UAS-ombRNAi *was overexpressed in the *en-Gal4 *domain. The disc is double stained with phalloidin (red) and anti-Omb (green) (B'). The fluorescence intensity distribution (inserted green curve in B') in a stripe of cells (orange box) along the A/P axis revealed the low residual staining in the *ombRNAi *territory. (C-E) Omb distribution in early, middle, and late third instar wing discs. Omb is graded throughout the third larval stage. Click here for file

Additional file 2 **Relative Omb expression in *act5C>tkv*^*QD *^clones and attenuation of overexpression by omb-RNAi co-expression**. (A) Lateral *tkv*^*QD *^clones (marked by the absence of CD2, green, arrowheads) up-regulate Omb (red) to a level comparable to central endogenous Omb. Disc shape and the endogenous Omb expression domain are contorted due to the proliferative effect of ectopic Dpp signaling and the disturbance of the Dpp gradient. (A') Fluorescence intensity was measured along the yellow angular line. (A'') Comparison of Omb expression in clones and in the center of wild type discs does not show a significant difference. (B) x-y confocal section of *act5C>(tkvQD+ombRNAi) *wing disc. The periphery of the wing pouch and retracting cell clones are visualized by red phalloidin staining. A lateral clone in which retraction is suppressed by U*AS*-*ombRNAi *co-expression is circled by a dotted line (arrowhead). This clone is shown in a x-z section in Fig. 2F. Click here for file

Additional file 3 **Influence of *tkv*^*QD *^clone size on the position of the apico-basal retraction**. (A) Large *tkv*^*QD *^clones (marked by co-expression of CD8-GFP, arrowheads) retract cells at the clonal border. (B) Small *tkv*^*QD *^clones (marked by the absence of CD2) retract cells in the clonal center. (A' and B') x-z scans from the panels above. Clones are marked by GFP expression (green in A and A') or strong anti-Omb staining (blue in B'). Click here for file

Additional file 4 **Graded apical microtubule web density in the wing imaginal disc. Confocal micrograph of a cryostat x-z-sections of an embedded wing disc**. The arrowheads indicate α-tubulin enrichment (green) both in the overlying peripodial membrane and in the AMW of the main epithelium. AMW density is attenuated towards the lateral edges of the wing pouch which are marked by *brk-lacZ *epression (red). Click here for file

Additional file 5 **Cellular retraction in *omb *clones is independent of cell death**. (A, A', and B) Activated caspase-3 staining (green) does not correlate with *omb *clones marked by loss of Omb staining (blue). (A') Higher magnification of boxed section in (A). Although Caspase-3 positive cells can be present at the clonal border (arrow), many retracting clones show no evidence of cell death (arrowheads). (B) Cellular retraction in *omb *clones without activation of caspase-3 (x-z section). (C) Staining against DE-cadherin (red) shows that retracting cells (marked by absence of GFP) retained apical contact among themselves and to the surrounding phenotypically wild type cells. Caspase-3 (blue) is not activated in retracting cells. (D and E) *omb *clones (marked by loss of Omb, blue, dotted outline) continue to undergo mitosis as revealed by anti-PH3 staining (green, arrowheads). (E) x-z section. Click here for file

Additional file 6 **Strong reduction of Omb expression by ombRNAi**. Plot profile of anti-Omb intensity in Fig. 3B. Note that the image was rotated 17° CCW using Photoshop 6.0 program. (A) A more apical confocal section of phalloidin staining (red, to show the accumulation of phalloidin in *ombRNAi *clones) and a middle section of Omb staining (green, to show the loss of Omb staining in *ombRNAi *clones) were merged. (A') The plot profile of anti-Omb staining in a stripe of cells (orange box) shows a graded distribution disrupted by the *ombRNAi *clone (arrow). Click here for file

Additional file 7 **Repression of cell death does not prevent cellular retraction caused by *omb *overexpression**. (A) overexpression of *omb *(bright green anti-Omb staining) induces cell death (separated red caspase-3 staining in A'). Repression of cell death by co-expressing a dominant negative form of *bsk *(B) or P35 (C) does not prevent the cellular retraction (arrow heads). (C') x-z scan through clone marked by arrow in C. Click here for file
