# Supplementary figures and images for: Spatial discontinuity of Optomotor-blind expression in the Drosophila wing imaginal disc disrupts epithelial architecture and promotes cell sorting
Source: BMC Dev Biol. 2010 Feb 23;10:23. doi: 10.1186/1471-213X-10-23 (PMC2838827; doi:10.1186/1471-213X-10-23)

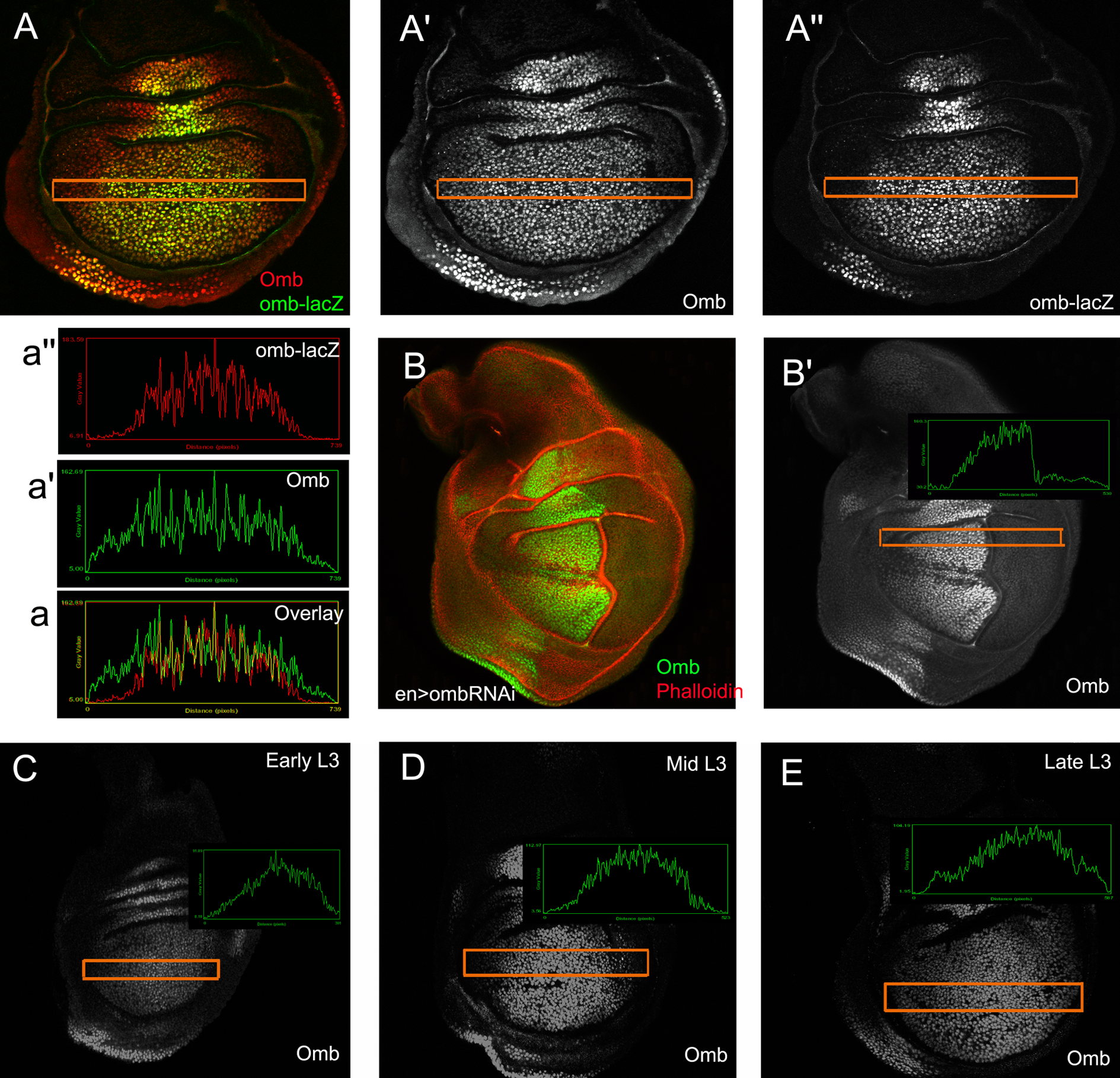

Supplement: Additional file 1 — Difference in the steepness of graded expression between Omb immunofluorescence and the omb-lacZ enhancer trap line ombP1. (A) ombP1 disc double stained with anti-Omb (A') and anti-β-galactosidase (A''). The fluorescence intensity distributions in stripes of cells (orange boxes) along the A/P axis were measured using the Image-J program and are shown in a-a". (B) UAS-ombRNAi was overexpressed in the en-Gal4 domain. The disc is double stained with phalloidin (red) and anti-Omb (green) (B'). The fluorescence intensity distribution (inserted green curve in B') in a stripe of cells (orange box) along the A/P axis revealed the low residual staining in the ombRNAi territory. (C-E) Omb distribution in early, middle, and late third instar wing discs. Omb is graded throughout the third larval stage. [file 1471-213X-10-23-S1.TIFF]

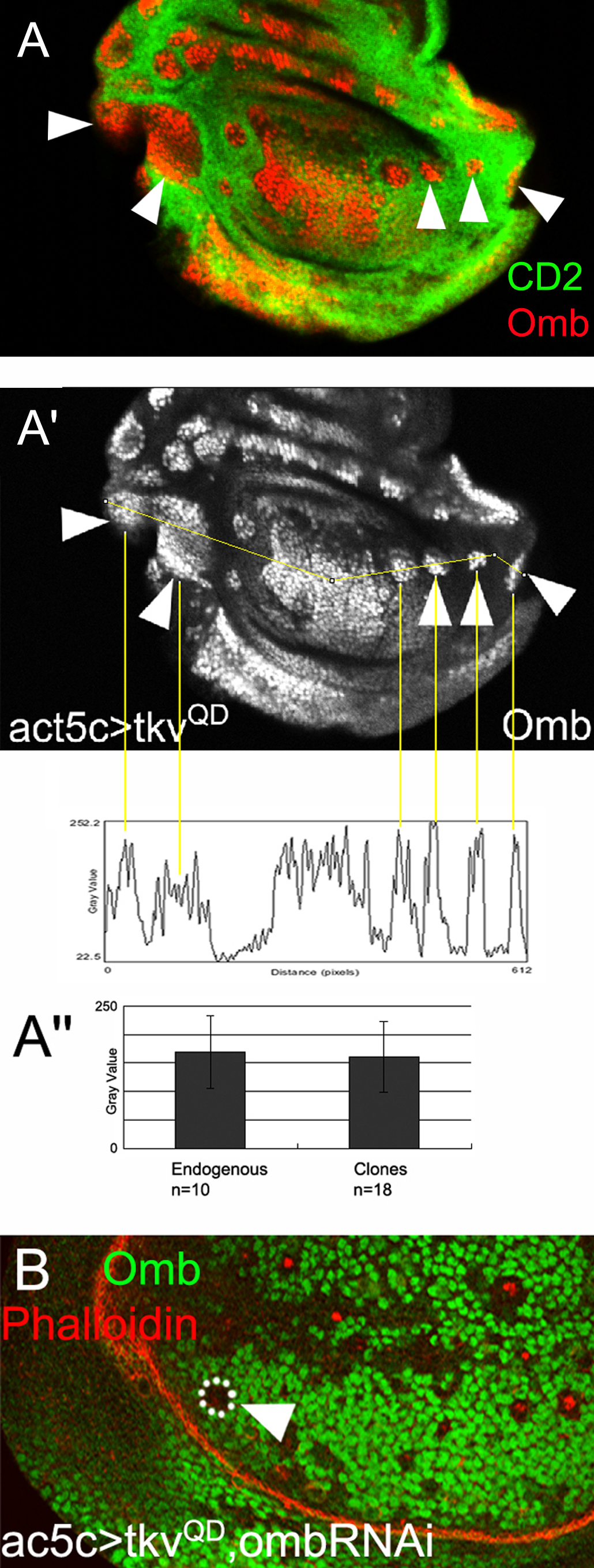

Supplement: Additional file 2 — Relative Omb expression in act5C>tkvQD clones and attenuation of overexpression by omb-RNAi co-expression. (A) Lateral tkvQD clones (marked by the absence of CD2, green, arrowheads) up-regulate Omb (red) to a level comparable to central endogenous Omb. Disc shape and the endogenous Omb expression domain are contorted due to the proliferative effect of ectopic Dpp signaling and the disturbance of the Dpp gradient. (A') Fluorescence intensity was measured along the yellow angular line. (A'') Comparison of Omb expression in clones and in the center of wild type discs does not show a significant difference. (B) x-y confocal section of act5C>(tkvQD+ombRNAi) wing disc. The periphery of the wing pouch and retracting cell clones are visualized by red phalloidin staining. A lateral clone in which retraction is suppressed by UAS-ombRNAi co-expression is circled by a dotted line (arrowhead). This clone is shown in a x-z section in Fig. 2F. [file 1471-213X-10-23-S2.TIFF]

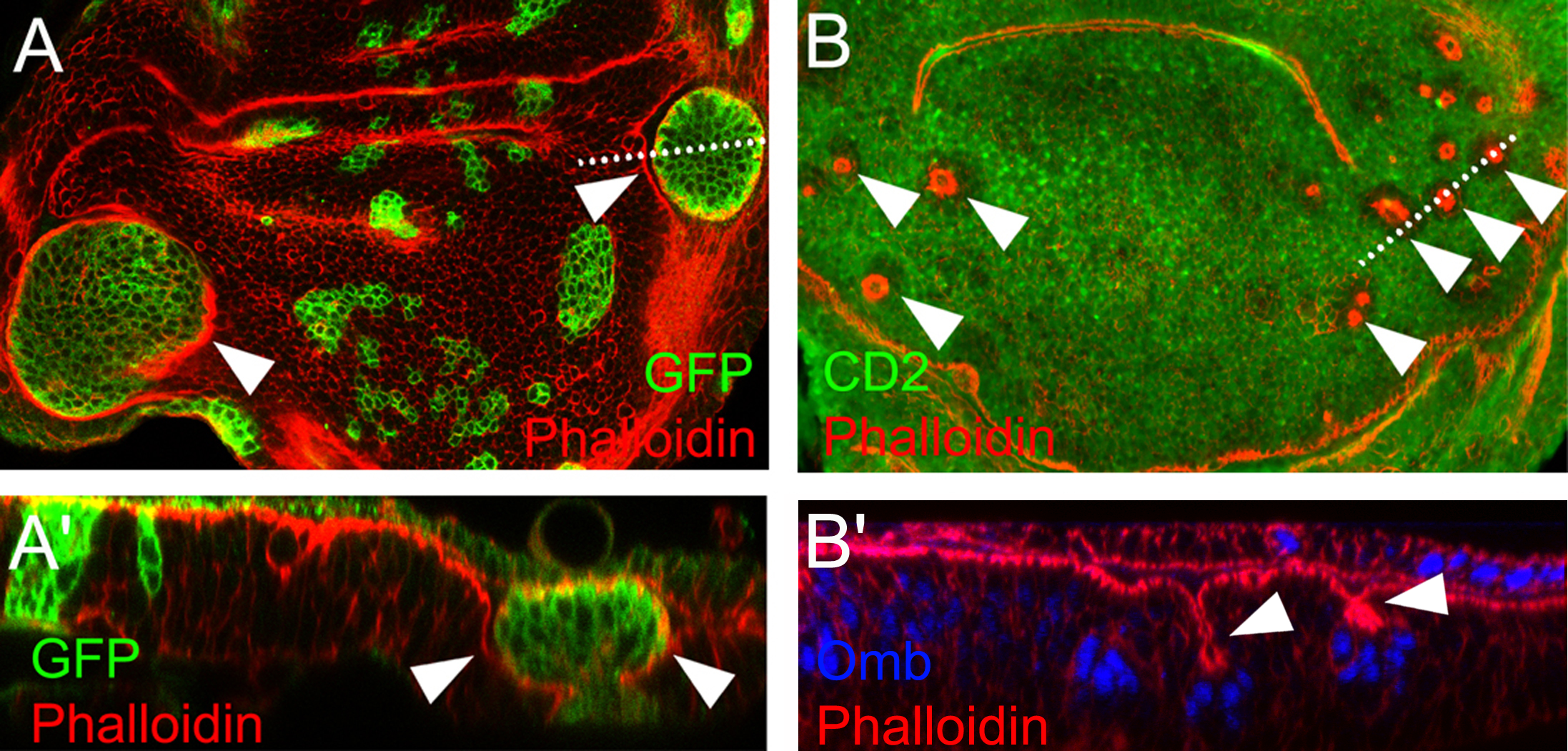

Supplement: Additional file 3 — Influence of tkvQD clone size on the position of the apico-basal retraction. (A) Large tkvQD clones (marked by co-expression of CD8-GFP, arrowheads) retract cells at the clonal border. (B) Small tkvQD clones (marked by the absence of CD2) retract cells in the clonal center. (A' and B') x-z scans from the panels above. Clones are marked by GFP expression (green in A and A') or strong anti-Omb staining (blue in B'). [file 1471-213X-10-23-S3.TIFF]

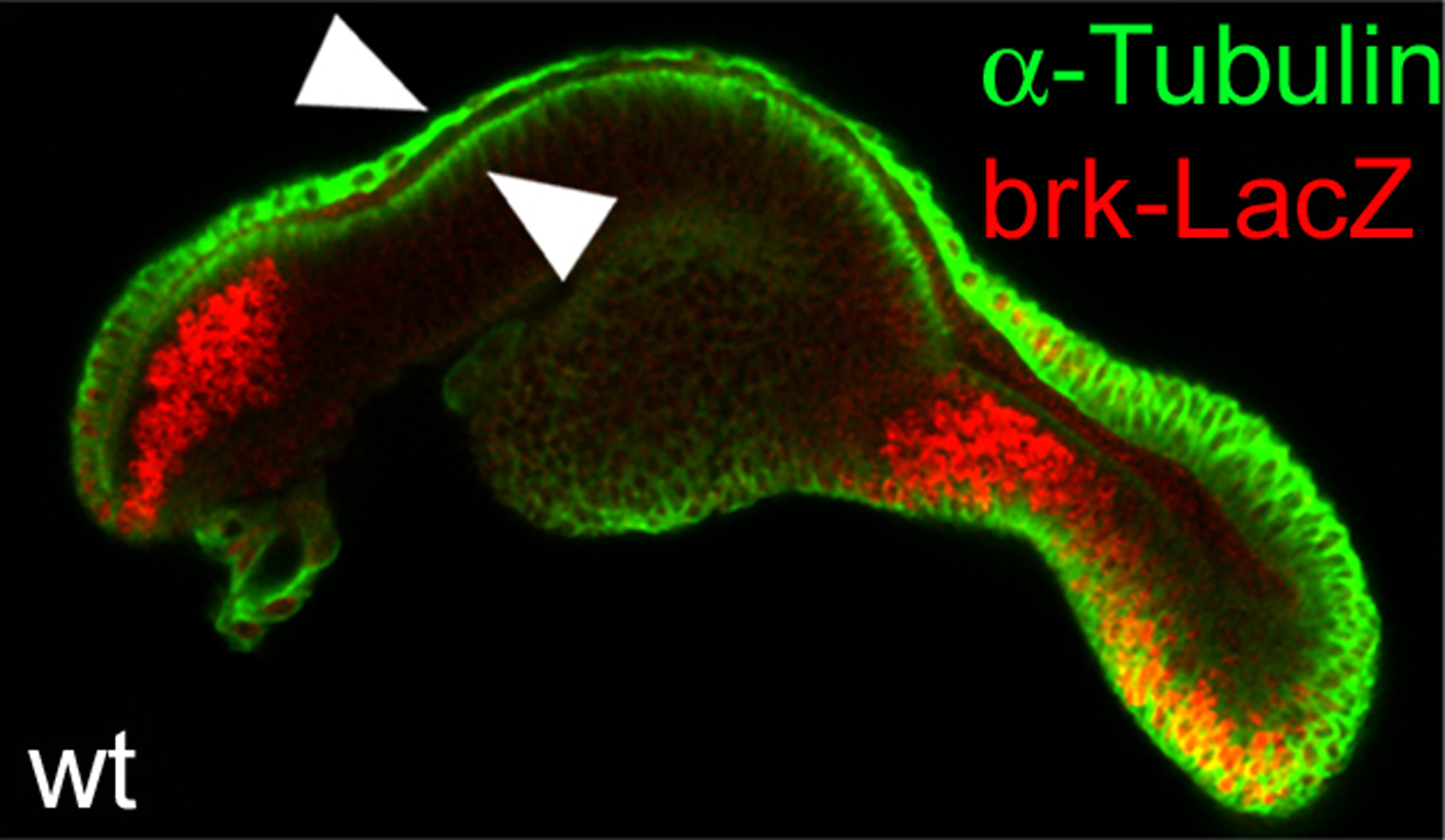

Supplement: Additional file 4 — Graded apical microtubule web density in the wing imaginal disc. Confocal micrograph of a cryostat x-z-sections of an embedded wing disc. The arrowheads indicate α-tubulin enrichment (green) both in the overlying peripodial membrane and in the AMW of the main epithelium. AMW density is attenuated towards the lateral edges of the wing pouch which are marked by brk-lacZ epression (red). [file 1471-213X-10-23-S4.TIFF]

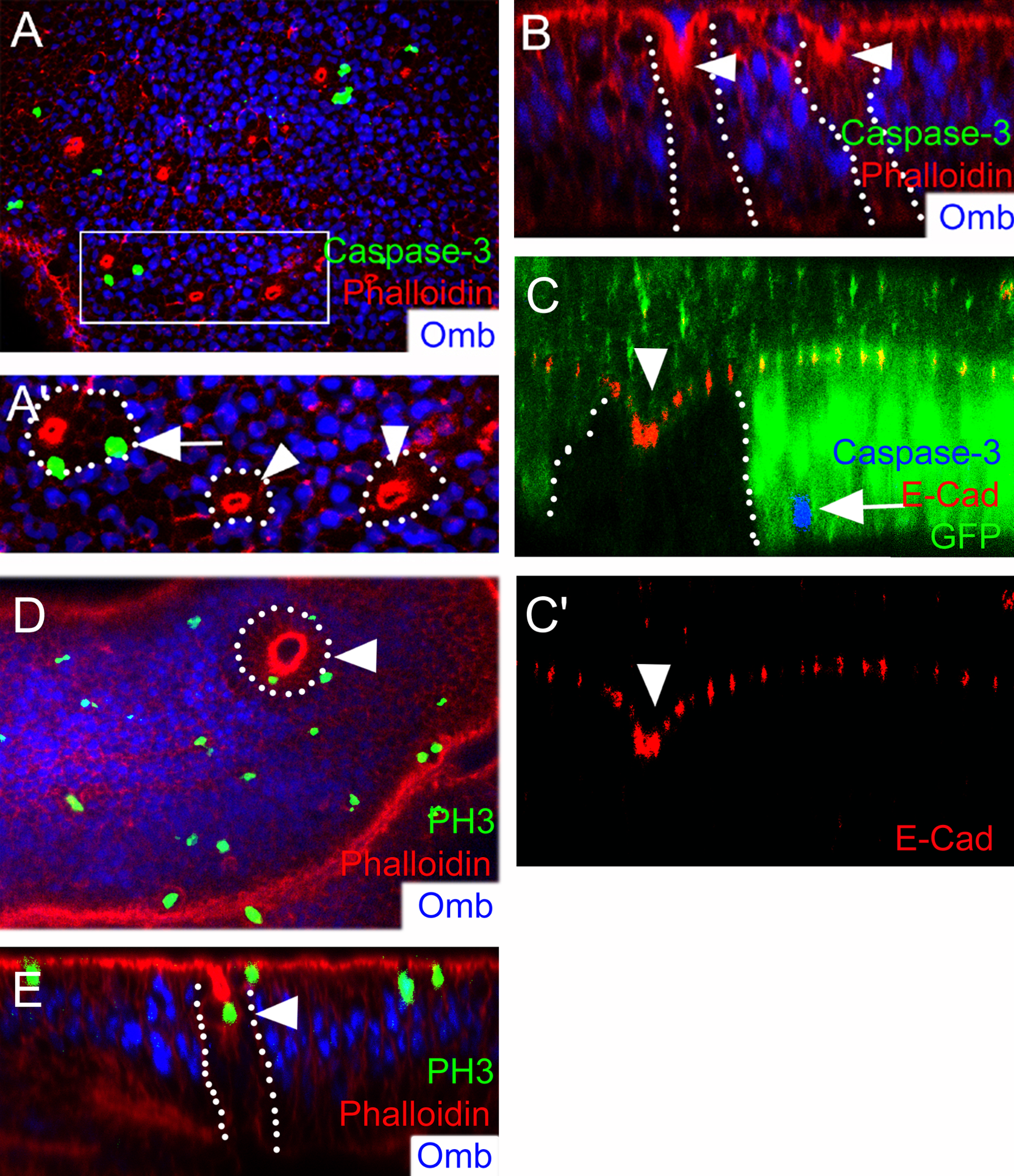

Supplement: Additional file 5 — Cellular retraction in omb clones is independent of cell death. (A, A', and B) Activated caspase-3 staining (green) does not correlate with omb clones marked by loss of Omb staining (blue). (A') Higher magnification of boxed section in (A). Although Caspase-3 positive cells can be present at the clonal border (arrow), many retracting clones show no evidence of cell death (arrowheads). (B) Cellular retraction in omb clones without activation of caspase-3 (x-z section). (C) Staining against DE-cadherin (red) shows that retracting cells (marked by absence of GFP) retained apical contact among themselves and to the surrounding phenotypically wild type cells. Caspase-3 (blue) is not activated in retracting cells. (D and E) omb clones (marked by loss of Omb, blue, dotted outline) continue to undergo mitosis as revealed by anti-PH3 staining (green, arrowheads). (E) x-z section. [file 1471-213X-10-23-S5.TIFF]

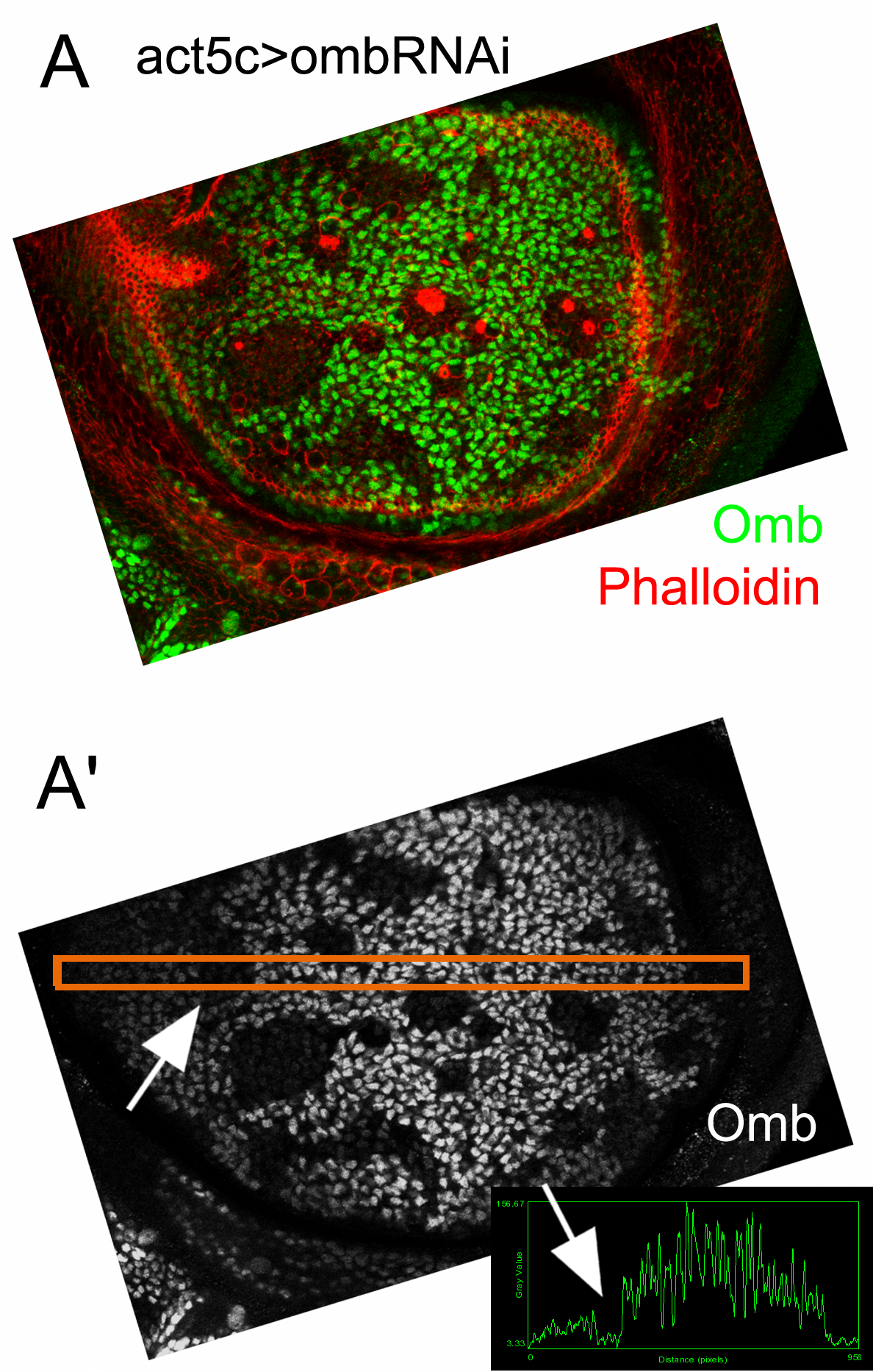

Supplement: Additional file 6 — Strong reduction of Omb expression by ombRNAi. Plot profile of anti-Omb intensity in Fig. 3B. Note that the image was rotated 17° CCW using Photoshop 6.0 program. (A) A more apical confocal section of phalloidin staining (red, to show the accumulation of phalloidin in ombRNAi clones) and a middle section of Omb staining (green, to show the loss of Omb staining in ombRNAi clones) were merged. (A') The plot profile of anti-Omb staining in a stripe of cells (orange box) shows a graded distribution disrupted by the ombRNAi clone (arrow). [file 1471-213X-10-23-S6.TIFF]

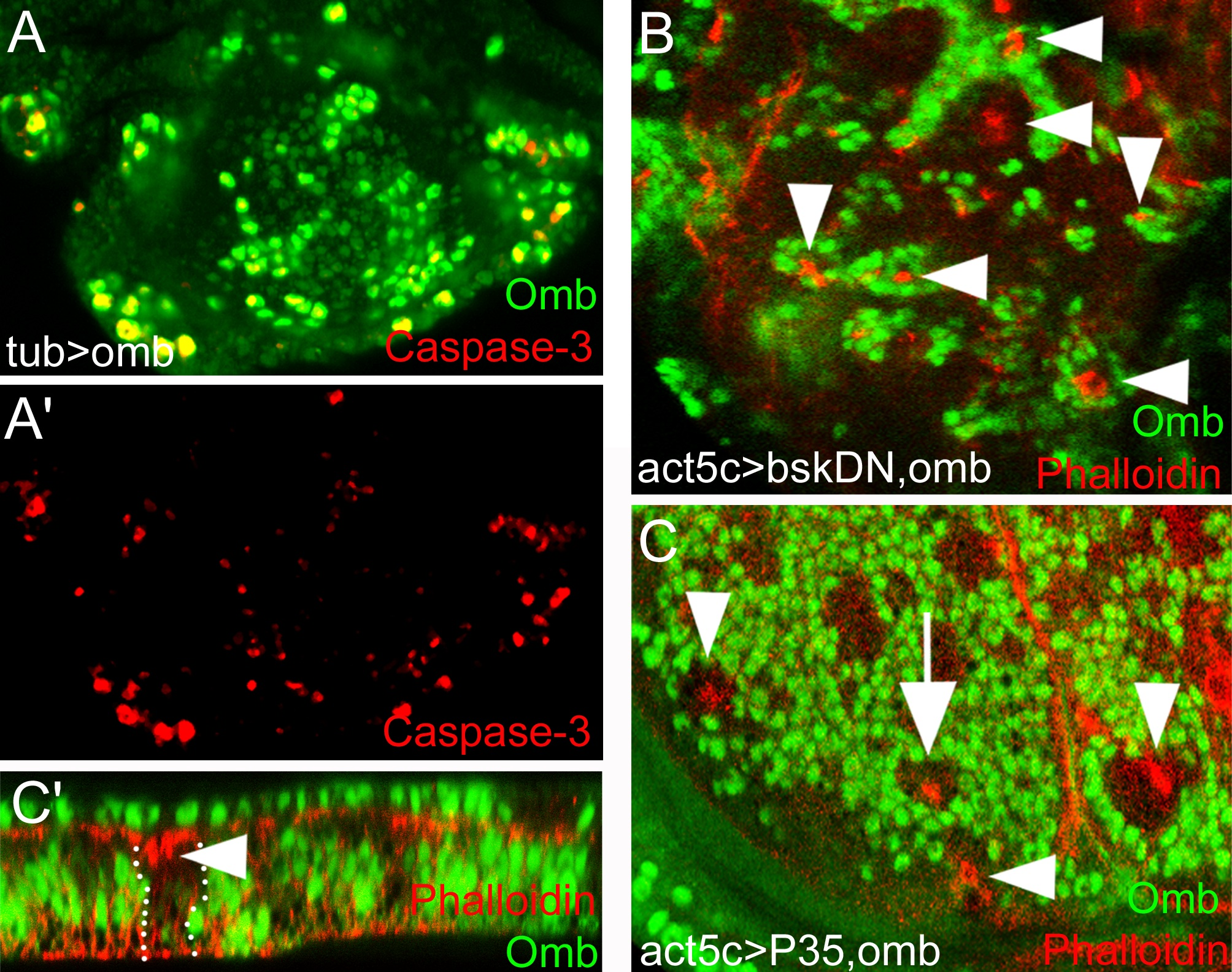

Supplement: Additional file 7 — Repression of cell death does not prevent cellular retraction caused by omb overexpression. (A) overexpression of omb (bright green anti-Omb staining) induces cell death (separated red caspase-3 staining in A'). Repression of cell death by co-expressing a dominant negative form of bsk (B) or P35 (C) does not prevent the cellular retraction (arrow heads). (C') x-z scan through clone marked by arrow in C. [file 1471-213X-10-23-S7.TIFF]
